# Plume moths (Lepidoptera, Pterophoridae) reared from the Chilean endemic *Steviaphilippiana* (Asteraceae)

**DOI:** 10.3897/BDJ.10.e94358

**Published:** 2022-09-28

**Authors:** Héctor A. Vargas

**Affiliations:** 1 Universidad de Tarapacá, Facultad de Ciencias Agronómicas, Departamento de Recursos Ambientales, Arica, Chile Universidad de Tarapacá, Facultad de Ciencias Agronómicas, Departamento de Recursos Ambientales Arica Chile

**Keywords:** arid environments, central Andes, flower-feeding larvae, leaf-feeding larvae, mitochondrial DNA

## Abstract

**Background:**

The micromoth fauna of the arid environments of the western slopes of central Andes remains poorly explored. Plants native to this area host overlooked species. A survey for micromoth larvae on the Chilean endemic *Steviaphilippiana* Hieron. (Asteraceae) was performed.

**New information:**

The first records of plume moths (Lepidoptera, Pterophoridae) associated with *S.philippiana* are provided. Adults of *Adainajobimi* Vargas, 2020 and a new species of *Oidaematophorus* Wallengren, 1862 were reared from larvae collected on inflorescences and leaves, respectively. *Oidaematophorusandresi* sp. n. is described and illustrated. A phylogenetic analysis of mitochondrial DNA sequences clustered each of the two plume moths with the type species of its respective genus. These records expand the host plant range of *A.jobimi* and add a second species of *Oidaematophorus* to the Chilean fauna of plume moths.

## Introduction

Along the altitudinal gradient of the northernmost part of Chile, extending from sea level to the highlands of the Andes, the highest plant diversity occurs in a narrow altitudinal belt around 3500 m elevation (Arroyo et al. 1988, Rundel et al. 2003, Moreira-Muñoz et al. 2016). The micromoth fauna of this altitudinal belt remains poorly explored. However, recent studies revealed that native plants host previously overlooked species, including representatives of the family Pterophoridae (Vargas et al. 2020), suggesting that surveys for larvae on these plants could help to improve the understanding of the micromoth diversity of this area.

The Chilean endemic *Steviaphilippiana* Hieron. (Asteraceae) is a morphologically variable shrub or subshrub whose geographic distribution is restricted to two disjunct areas in the north of the country, one at high elevations on the western slopes of the Andes between 18 and 19°S, the other near sea level on the coast of the Atacama Desert between 22 and 26°S (Gutiérrez et al. 2016). Surveys for micromoth larvae in the Andes revealed that two species of plume moths belonging to two genera of the tribe Oidaematophorini (Pterophorinae) use *S.philippiana* as a host. The aim of this study is to provide these records, including the description of a new species of *Oidaematophorus* Wallengren, 1862. Furthermore, as some genera of Oidaematophorini have remarkably similar morphology (Gielis 2011), the generic assignment of the two species reared from *S.philippiana* was assessed using phylogenetic analysis of mitochondrial DNA sequences.

## Materials and methods

The study site is about 2 km south of Socoroma Village (18°16’42’’S, 69°34’15’’W) in the Parinacota Province of northern Chile, at about 3400 m elevation on the western slopes of the Andes. It has a tropical xeric climate with seasonal rains concentrated mainly in summer (Luebert and Pliscoff 2006). Mature plume moth larvae were collected on *S.philippiana* in March 2021 and April 2022. The collected larvae were placed in plastic vials with inflorescences or leaves, depending upon which plant organ they were feeding on in the field with a paper towel at the bottom. The emerged adults were mounted following standard procedures. For genitalia dissection, the abdomen was removed and placed in hot 10% potassium hydroxide (KOH) for a few minutes. The genitalia were stained with Eosin Y and Chlorazol Black and mounted on slides with Euparal. Photos of the adults were taken with a Sony CyberShot DSC-HX200V digital camera. Photos of the genitalia were taken with a Leica MC170 HD digital camera attached to a Leica DM1000 LED light microscope. Each image of the genitalia was constructed with 3–10 photos assembled with the software Helicon Focus 8. The specimens studied are deposited in the “Colección Entomológica de la Universidad de Tarapacá” (IDEA), Arica, Chile.

Two pupae reared from larvae collected on inflorescences and two legs from a female and a male adult reared from larvae collected on leaves were used for DNA extraction with the QIAamp Fast DNA Tissue Kit, following the manufacturer’s instructions. As genitalia morphology suggested that the adults reared from inflorescences belong to *Adainajobimi* Vargas, 2020, whose original description was based on specimens reared from inflorescences of *Baccharisalinfolia* Meyen & Walp. (Asteraceae) (Vargas 2020), DNA was also extracted from two pupae of *A.jobimi* reared from larvae collected on this plant in the Copaquilla ravine (18°23’55’’S, 69°37’49’’W) at about 2800 m elevation, 12 kilometres south of the study site. Genomic DNA was sent to Macrogen Inc. (Seoul, South Korea) for purification, PCR amplification and sequencing of the barcode region (Hebert et al. 2003) using the primers LCO1490 and HCO2198 (Folmer et al. 1994). The PCR programme was 5 min at 94°C, 35 cycles of 30 s at 94°C, 30 s at 47°C, 1 min at 72°C and a final elongation step of 10 min at 72°C. In order to assess the generic assignment of the plume moths reared from *S.philippiana*, the sequences obtained were submitted to a Maximum Likelihood (ML) phylogenetic analysis. As shown in Table 1, the alignment included sequences of the type species of the genera of Oidaematophorini represented in the Neotropical Region (*Adaina* Tutt, 1905, *Emmelina* Tutt, 1905, *Hellinsia* Tutt, 1905 and *Oidaematophorus*) and three outgroup genera of Platyptiliini (*Lioptilodes* Zimmerman, 1958, *Platyptilia* Hübner, [1825] and *Stenoptilia* Hübner, [1825]) downloaded from BOLD (Ratnasingham and Hebert 2007). The restriction of the taxon sampling of Oidaematophorini to the type species of each genus was due to generic definitions being unstable, as evidenced by frequent changes of some species between genera (Gielis 1991, Gielis 2011, Gielis 2014). The software MEGA11 (Tamura et al. 2021) was used to perform sequence alignment with the ClustalW method and to determine genetic distance using the Kimura 2-Parameter (K2P) method. Before the ML analysis, the substitution saturation of the alignment was assessed with the Xia test, using the software DAMBE7 (Xia 2018). The ML analysis was performed with the software IQTREE 1.6.12 (Nguyen et al. 2015) in the web interface W-IQ-TREE (Trifinopoulos et al. 2016). Data were partitioned to codon position. ModelFinder (Kalyaanamoorthy et al. 2017) selected TN+F+I, F81+F+I and HKY+F+R2 as the best-fit models for 1st, 2nd and 3rd partitions, respectively. Branch support was assessed with 1,000 replications of the Shimodaira-Hasegawa-like approximate likelihood ratio test (SH-aLRT, Guindon et al. 2010) and ultrafast bootstrap (UFBoot, Hoang et al. 2017). The unrooted tree was visualised in FigTree (Rambaut 2014) to root on Platyptiliini.

## Taxon treatments

### Oidaematophorus andresi

Vargas sp. n.

A5C1E7A6-2BC0-5BBB-965B-875ECBAB6667

BBDAE182-9EEC-4D2D-AE82-A028D9188236

#### Materials

- **Type status:** Holotype. **Occurrence:** occurrenceID: 916B29C3-0CE3-58D3-A958-110823634B2F; sex: male; associatedSequences: GenBank: OP281687; **Taxon:** order: Lepidoptera; family: Pterophoridae; genus: Oidaematophorus; specificEpithet: *andresi*; taxonRank: species; nomenclaturalCode: ICZN; **Location:** continent: South America; country: Chile; stateProvince: Parinacota; locality: About 2 km south of Socoroma village; verbatimElevation: 3400 m; verbatimLatitude: 18°16’42’’S; verbatimLongitude: 69°34’15’’W; **Identification:** identifiedBy: Héctor A. Vargas; **Event:** samplingProtocol: One male adult emerged May 2022, reared from larva collected on *Steviaphilippiana* April 2022; year: 2022; verbatimEventDate: May 2022; **Record Level:** type: PhysicalObject; language: en; institutionCode: IDEA; ownerInstitutionCode: IDEA-LEPI-2022-007, genitalia slide HAV-1538; basisOfRecord: PreservedSpecimen
- **Type status:** Paratype. **Occurrence:** occurrenceID: 3037D049-427A-586C-B398-3E21440B8BB9; sex: female; associatedSequences: GenBank: OP281688; **Taxon:** order: Lepidoptera; family: Pterophoridae; genus: Oidaematophorus ; specificEpithet: *andresi*; taxonRank: species; nomenclaturalCode: ICZN; **Location:** continent: South America; country: Chile; stateProvince: Parinacota; locality: About 2 km south of Socoroma village; verbatimElevation: 3400 m; verbatimLatitude: 18°16’42’’S; verbatimLongitude: 69°34’15’’W; **Identification:** identifiedBy: Héctor A. Vargas; **Event:** samplingProtocol: One female adult emerged May 2022, reared from larva collected on *Steviaphilippiana* April 2022; year: 2022; verbatimEventDate: May 2022; **Record Level:** type: PhysicalObject; language: en; institutionCode: IDEA; ownerInstitutionCode: IDEA-LEPI-2022-008, genitalia slide HAV-1539; basisOfRecord: PreservedSpecimen
- **Type status:** Paratype. **Occurrence:** occurrenceID: 15EE9771-54A9-5362-813D-9ACF4D89DE8A; sex: male; **Taxon:** order: Lepidoptera; family: Pterophoridae; genus: Oidaematophorus ; specificEpithet: *andresi*; taxonRank: species; nomenclaturalCode: ICZN; **Location:** continent: South America; country: Chile; stateProvince: Parinacota; locality: About 2 km south of Socoroma village; verbatimElevation: 3400 m; verbatimLatitude: 18°16’42’’S; verbatimLongitude: 69°34’15’’W; **Identification:** identifiedBy: Héctor A. Vargas; **Event:** samplingProtocol: One male adult emerged May 2022, reared from larva collected on *Steviaphilippiana* April 2022; year: 2022; verbatimEventDate: May 2022; **Record Level:** type: PhysicalObject; language: en; institutionCode: IDEA; ownerInstitutionCode: IDEA-LEPI-2022-009, genitalia slide HAV-1551; basisOfRecord: PreservedSpecimen
- **Type status:** Paratype. **Occurrence:** occurrenceID: 8A8880FD-CCE2-5EE5-ABB4-D42452D35139; sex: male; **Taxon:** order: Lepidoptera; family: Pterophoridae; genus: Oidaematophorus ; specificEpithet: *andresi*; taxonRank: species; nomenclaturalCode: ICZN; **Location:** continent: South America; country: Chile; stateProvince: Parinacota; locality: About 2 km south of Socoroma village; verbatimElevation: 3400 m; verbatimLatitude: 18°16’42’’S; verbatimLongitude: 69°34’15’’W; **Identification:** identifiedBy: Héctor A. Vargas; **Event:** samplingProtocol: One male adult emerged May 2022, reared from larva collected on *Steviaphilippiana* April 2022; year: 2022; verbatimEventDate: May 2022; **Record Level:** type: PhysicalObject; language: en; institutionCode: IDEA; ownerInstitutionCode: IDEA-LEPI-2022-010, genitalia slide HAV-1556; basisOfRecord: PreservedSpecimen

#### Description

**Figure 1.**
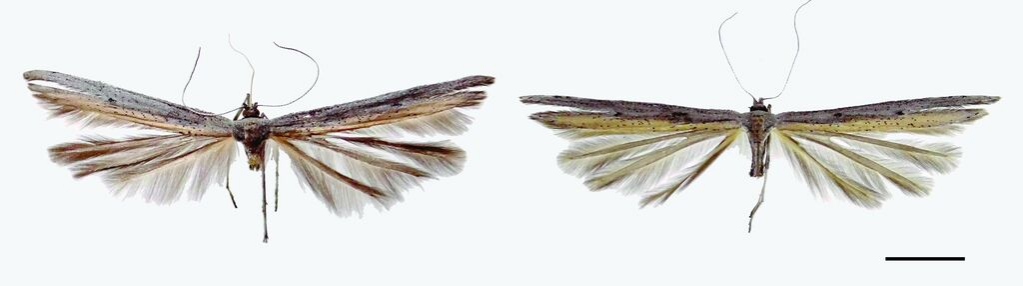
Habitus of *Oidaematophorusandresi* sp. n. Left, male holotype (IDEA-LEPI-2022-007). Right, female paratype (IDEA-LEPI-2022-008).

Male and female (Fig. 1). Fore-wing length 13.5–14.1 mm.

Head. Vertex and frons mostly grey with scattered white scales. Occiput with erect, narrow, dark grey scales. Labial palpus with first and second segments white, third segment grey. Antenna filiform, about half the costa length, with grey and white scales.

Thorax. Mostly grey with scattered white, brown and black scales. Fore-leg coxa mostly grey with longitudinal row of black-tipped scales anteriorly; femur and tibia tarsus grey. Mid- and hind-leg grey. Fore-wing cleft origin at about 2/3 from wing base. Dorsal surface mostly grey, with a longitudinal yellowish-brown stripe along the anal margin from near the wing base to the complete second lobe; discal spot black; a black spot before cleft base; two black spots on costa near the middle of first lobe; scattered black scales near anal margin; fringe grey; ventral surface grey. Hind-wing dorsal and ventral surfaces and fringe grey.

Abdomen. Mostly grey with scattered white and brown scales.

**Figure 2.**
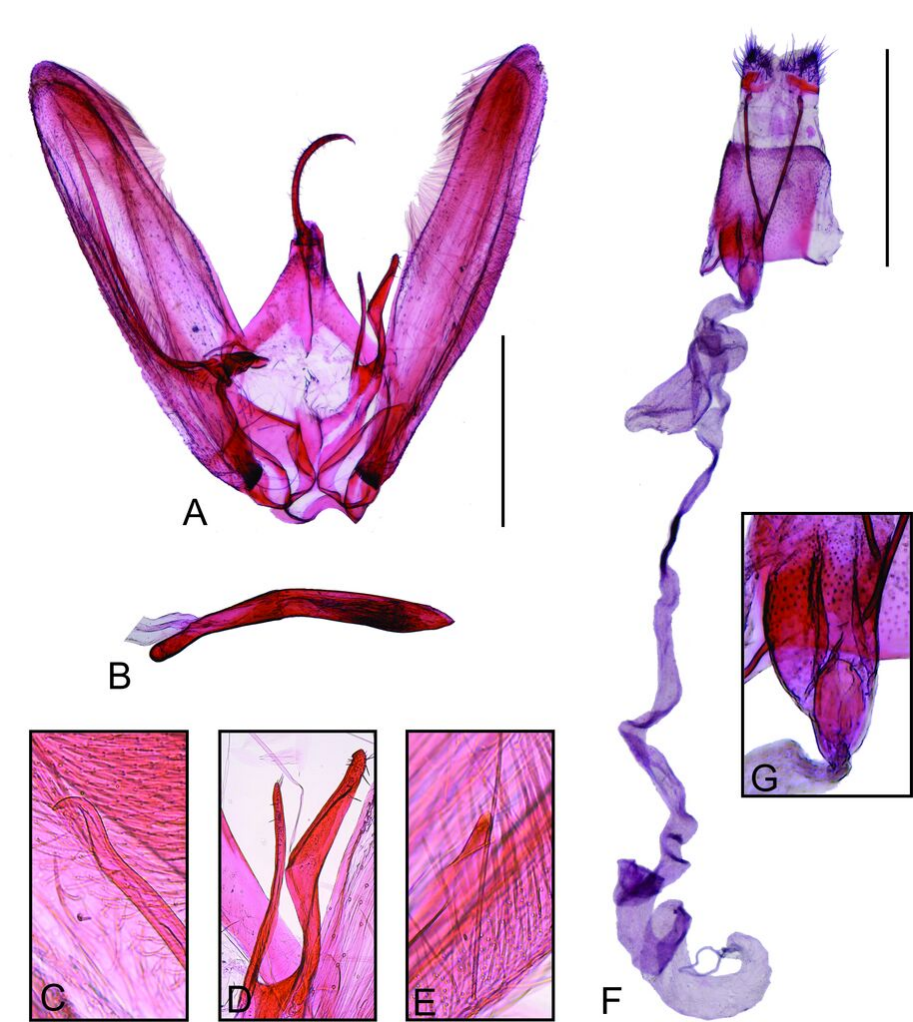
Genitalia of *Oidaematophorusandresi* sp. n. **A** Male genitalia in ventral view, phallus removed. **B** Phallus in lateral view. **C** Apex of the saccular spine of the left valva. **D** Anellus arms. **E** Dentate process of the sacculus of the right valva. **F** Female genitalia in ventral view. **G** Detail of the antrum. Male holotype IDEA-LEPI-2022-007, genitalia slide HAV-1538. Female paratype IDEA-LEPI-2022-008, genitalia slide HAV-1539. Scale bar 1 mm.

Male genitalia (Fig. 2). Tegumen bilobed; anterior margin with triangular projection medially. Uncus narrow, slender, curved, apex pointed. Vinculum narrow. Saccus slightly curved in the middle. Juxta asymmetrical, strongly curved to right, left margin more strongly sinuous than right margin, a narrow longitudinal membranous stripe along the middle almost reaching the base of anellus arms. Anellus arms asymmetrical; left arm narrow, slightly curved, with an apical row of small setae; right arm wider than left arm, strongly curved in the middle, with a small subapical projection, a few small setae near and a row of small setae on the opposite side. Valvae asymmetrical, each with a longitudinal fold in the middle and a group of hair-like scales arising basally on external side. Left valva slightly wider than right one; apex rounded; saccular process with a somewhat conical basal section and a slender saccular spine; saccular spine slightly longer than three fourths the costal margin length, basal fourth of saccular spine rounded towards ventral margin of valva, distal three-fourths straight, apex with hooked tip. Right valva with a single dentate process on the sacculus. Phallus cylindrical, curved, acute apex, vesica without cornuti.

Female genitalia (Fig. 2). Papilla analis short, posteriorly rounded, mostly slightly sclerotised, with a well-sclerotised band along anterior margin. Posterior apophysis (apex of the left posterior apophysis broken during mounting), narrow, rod-shaped, about four times the length of papilla analis, apex almost reaches the anterior margin of tergum VIII. Anterior apophysis from anterior vertex of tergum VIII, narrow, rod-shaped, about a half the length of papilla analis. Ostium bursae displaced to left. Antrum cup-shaped, wider posteriorly, mostly slightly sclerotised, with an oval-shaped sclerite near the junction with ductus bursae. Ductus bursae membranous, narrow, diameter about half of the widest part of antrum. Corpus bursae membranous, elongated, about three times the length of ductus bursae. Ductus seminalis from near the junction of ductus bursae with corpus bursae, about six times as long as corpus bursae, anterior part coiled.

#### Diagnosis

*Oidaematophorusandresi* sp. n. can be distinguished from other Neotropical representatives of the genus by the dorsal surface of the fore-wing mostly grey, with a longitudinal yellowish-brown stripe along the anal margin from near the wing base to the complete second lobe. The male genitalia of *O.andresi* sp. n. resemble those of *O.espeletiae*, Hernández, Fuentes, Fajardo & Matthews, 2014 from Colombia (Hernández et al. 2014) in having a hooked tip on the apex of the spine of the saccular process of the left valva. However, the dorsal surface of the fore-wing of *O.espeletiae* is mostly grey at base and mostly cream apically. Furthermore, the saccular process of the left valva is straight along a great part of its length with a short curved portion near the base and the right valva has a single dentate process on the sacculus in the male genitalia of *O.andresi* sp. n. In contrast, the saccular process of the left valva has a great curved portion and the right valva has two dentate processes on the sacculus in the male genitalia in *O.espeletiae*. In the female genitalia, the posteriorly wider cup-shaped antrum of *O.andresi* sp. n. contrasts with the anteriorly wider antrum of *O.espeletiae*. Furthermore, the antrum of *O.andresi* sp. n. has an oval-shaped sclerite near the junction with ductus bursae, which is absent in *O.espeletiae*.

#### Etymology

The name of the species is dedicated to Dr. Andrés Moreira-Muñoz, for his remarkable contributions to the biogeography and systematics of the Chilean flora.

#### Distribution

**Figure 3.**
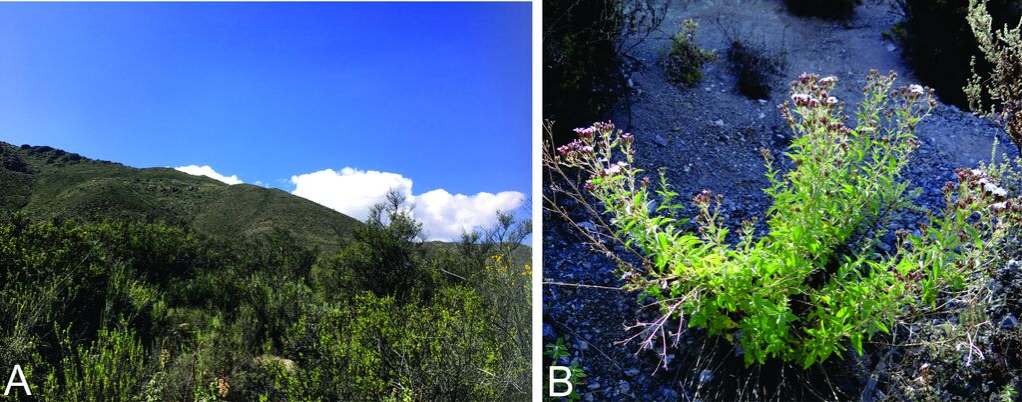
Habitat and host plant of *Oidaematophorusandresi* sp. n. **A** Habitat at the type locality, near Socoroma Village in the Andes of northern Chile; **B** *Steviaphilippiana* at the type locality.

*Oidaematophorusandresi* sp. n. is known only from the type locality, about 2 km south of Socoroma Village, at about 3400 m elevation on the western slopes of the Andes of northern Chile (Fig. 3).

#### Biology

The only host plant currently recorded for *O.andresi* sp. n. is *S.philippiana* (Fig. 3).

#### Taxon discussion

Species of *Oidaematophorus* are recognised by fore-wing venation with R1 absent, R2, R3, R4 and R5 separate, Cu1 from the posterior angle of the discal cell and Cu2 from the discal cell, mid-leg with scale bristles at base of spur pairs and female genitalia with bell- or widened funnel-shaped antrum (Gielis 2011). Ten described species of *Oidaematophorus* occur in the Neotropical Region (Gielis 2011, Gielis 2014, Hernández et al. 2014, Matthews et al. 2019, Ustjuzhanin et al. 2021b), only one of which, *O.pseudotrachyphloeus* Gielis, 2011, is known from Chile (Vargas 2021). Although eight species of the genus were recorded from this country earlier (Gielis 1991), these are currently included in *Hellinsia* (Gielis 2011). Accordingly, *O.andresi* sp. n. is the second representative of the genus confirmed from Chile. The two species from this country are easily recognised, based on wing pattern, as the fore-wing of *O.pseudotrachyphloeus* lacks the longitudinal yellowish-brown stripe along the anal margin typical of *O.andresi* sp. n. The genitalia also provide useful morphological characters in this case, as in *O.pseudotrachyphloeus* the male has the spine of the saccular process of the left valva strongly curved throughout its length and the female has asymmetrical anterior apophyses and ductus seminalis only slightly longer than the corpus bursae, in clear contrast to *O.andresi* sp. n. Although the host plant ranges of these two species must be explored further, the currently available records suggest that they use different host plants, because *O.pseudotrachyphloeus* has been reared only from *Ambrosiacumanensis* Kunth (Asteraceae) (Vargas 2021).

### Adaina jobimi

Vargas, 2020

CD911CDC-105F-5B2E-A87C-733EFF8EA67B

B1FB4445-979C-4C15-9D1D-E18D6C1E2412

#### Materials

- **Type status:** Other material. **Occurrence:** occurrenceID: 2D686B45-2BE2-54F3-AE29-A09AA30323EE; sex: male; otherCatalogNumbers: genitalia slide HAV-1557; **Taxon:** scientificNameID: urn:lsid:zoobank.org:act:B1FB4445-979C-4C15-9D1D-E18D6C1E2412; namePublishedInID: https://doi.org/10.3897/nl.43.57965; order: Lepidoptera; family: Pterophoridae; genus: Adaina; specificEpithet: jobimi; taxonRank: species; scientificNameAuthorship: Vargas, 2020; **Location:** continent: South America; country: Chile; stateProvince: Parinacota; locality: About 2 km south of Socoroma village; verbatimElevation: 3400 m; verbatimLatitude: 18°16’42’’S; verbatimLongitude: 69°34’15’’W; **Identification:** identifiedBy: Héctor A. Vargas; **Event:** samplingProtocol: One male adult emerged April 2021, reared from larva collected on *Steviaphilippiana* March 2021; verbatimEventDate: April 2021; **Record Level:** type: PhysicalObject; language: en; institutionCode: IDEA; basisOfRecord: PreservedSpecimen
- **Type status:** Other material. **Occurrence:** occurrenceID: F214C268-4C4E-529E-8CD1-89325ABBE667; sex: male; otherCatalogNumbers: genitalia slide HAV-1558; **Taxon:** scientificNameID: urn:lsid:zoobank.org:act:B1FB4445-979C-4C15-9D1D-E18D6C1E2412; namePublishedInID: https://doi.org/10.3897/nl.43.57965; order: Lepidoptera; family: Pterophoridae; genus: Adaina; specificEpithet: jobimi; taxonRank: species; scientificNameAuthorship: Vargas, 2020; **Location:** continent: South America; country: Chile; stateProvince: Parinacota; locality: About 2 km south of Socoroma village; verbatimElevation: 3400 m; verbatimLatitude: 18°16’42’’S; verbatimLongitude: 69°34’15’’W; **Identification:** identifiedBy: Héctor A. Vargas; **Event:** samplingProtocol: One male adult emerged April 2021, reared from larva collected on *Steviaphilippiana* March 2021; verbatimEventDate: April 2021; **Record Level:** type: PhysicalObject; language: en; institutionCode: IDEA; basisOfRecord: PreservedSpecimen
- **Type status:** Other material. **Occurrence:** occurrenceID: B9926EE2-1FB3-5F35-8AD6-12CA268EDFB4; sex: female; otherCatalogNumbers: genitalia slide HAV-1559; **Taxon:** scientificNameID: urn:lsid:zoobank.org:act:B1FB4445-979C-4C15-9D1D-E18D6C1E2412; namePublishedInID: https://doi.org/10.3897/nl.43.57965; order: Lepidoptera; family: Pterophoridae; genus: Adaina; specificEpithet: jobimi; taxonRank: species; scientificNameAuthorship: Vargas, 2020; **Location:** continent: South America; country: Chile; stateProvince: Parinacota; locality: About 2 km south of Socoroma village; verbatimElevation: 3400 m; verbatimLatitude: 18°16’42’’S; verbatimLongitude: 69°34’15’’W; **Identification:** identifiedBy: Héctor A. Vargas; **Event:** samplingProtocol: One female adult emerged April 2021, reared from larva collected on *Steviaphilippiana* March 2021; verbatimEventDate: April 2021; **Record Level:** type: PhysicalObject; language: en; institutionCode: IDEA; basisOfRecord: PreservedSpecimen
- **Type status:** Other material. **Occurrence:** occurrenceID: 18D1896C-0B5C-511A-957A-687DB11CCF6A; sex: female; otherCatalogNumbers: genitalia slide HAV-1560; **Taxon:** scientificNameID: urn:lsid:zoobank.org:act:B1FB4445-979C-4C15-9D1D-E18D6C1E2412; namePublishedInID: https://doi.org/10.3897/nl.43.57965; order: Lepidoptera; family: Pterophoridae; genus: Adaina; specificEpithet: jobimi; taxonRank: species; scientificNameAuthorship: Vargas, 2020; **Location:** continent: South America; country: Chile; stateProvince: Parinacota; locality: About 2 km south of Socoroma village; verbatimElevation: 3400 m; verbatimLatitude: 18°16’42’’S; verbatimLongitude: 69°34’15’’W; **Identification:** identifiedBy: Héctor A. Vargas; **Event:** samplingProtocol: One female adult emerged April 2021, reared from larva collected on *Steviaphilippiana* March 2021; verbatimEventDate: April 2021; **Record Level:** type: PhysicalObject; language: en; institutionCode: IDEA; basisOfRecord: PreservedSpecimen

#### Taxon discussion

Host plant records available for *Adaina* indicate that a single species may be able to feed on several Asteraceae belonging to one or more genera (Landry and Gielis 1992, Matthews and Lott 2005). *Baccharisalnifolia* Meyen & Walp. (Asteraceae) was the only host plant previously known for *A.jobimi* (Vargas 2020). Accordingly, rearing from *S.philippiana* adds a new host plant record and suggests that this plume moth is able to use distantly-related members of Asteraceae. As this plant family is well represented in the study area (Moreira-Muñoz et al. 2016), further surveys would be needed to know the complete host plant range of *A.jobimi*.

## Analysis

**Figure 4.**
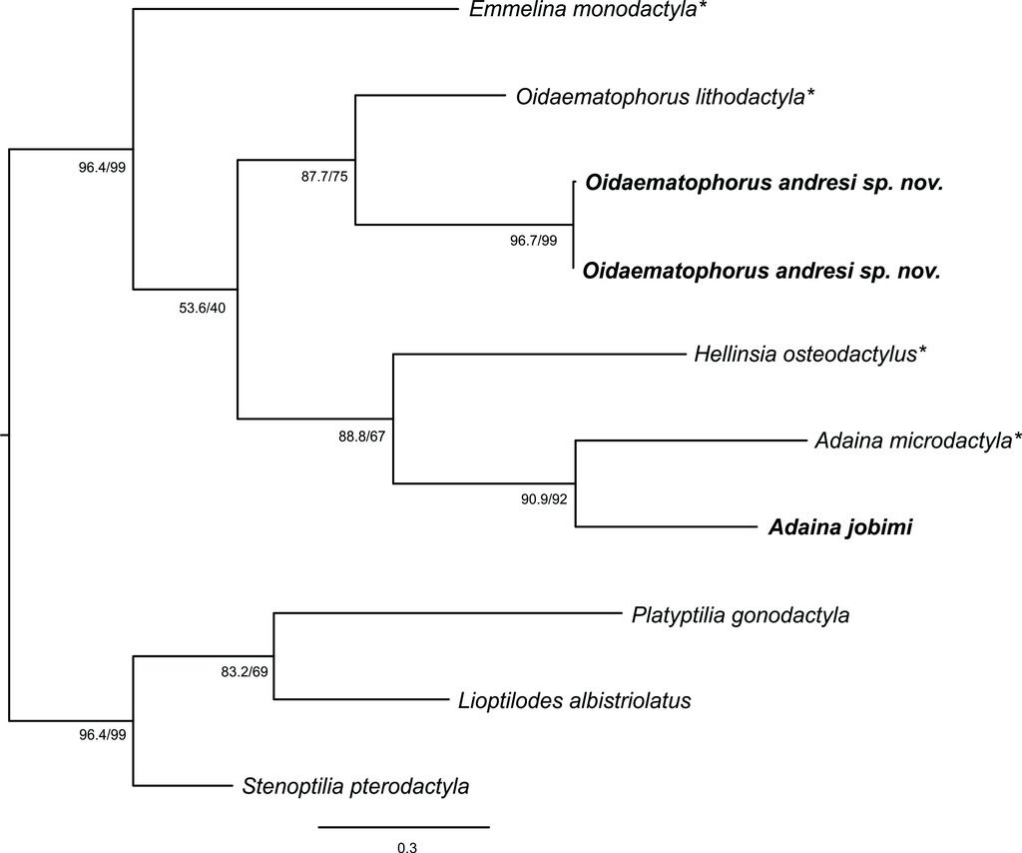
Maximum Likelihood tree of the plume moths reared from *Steviaphilippiana* (bold) and the type species (asterisks) of Oidaematophorini genera represented in the Neotropical Region, based on mitochondrial DNA sequences. Numbers indicate SH-aLRT/UFBoot values (1000 replicates).

Four identical DNA barcode sequences were obtained from the pupae of *A.jobimi* reared from larvae collected on *S.philippiana* (GenBank accessions OP281683, OP281684) and *B.alnifolia* (OP281685, OP281686), confirming the morphological identification. Two DNA barcode sequences (OP281687, OP281688) with 0.3% (K2P) distance between them were obtained from the adults of *O.andresi* sp. n. The alignment of ten sequences of 657 bp length was suitable for phylogenetic analysis, as no evidence of stop codons or substitution saturation (ISS < ISS.C; p < 0.001) was detected. The sequences of the two species were clustered with the type species of their respective genus, *Adainamicrodactyla* (Hübner, [1813]) and *Oidaematophoruslithodactyla* (Treitschke, 1833), in the ML tree (Fig. 4). Genetic distance was 9.6% between *A.jobimi* and *A.microdactyla* and 10.9–11.0% between *O.andresi* sp. n. and *O.lithodactyla*.

## Discussion

Asteraceae is one of the main host families of Pterophoridae and even a single species of this plant family can support multiple lineages of plume moths (Matthews and Lott 2005). In the present study, surveys for lepidopteran larvae on the endemic *S.philippiana* in the Andes of northern Chile enabled the rearing of two species of the tribe Oidaematophorini, *A.jobimi* and *O.andresi* sp. n. This discovery highlights the importance of surveys on native plants to improve the knowledge of the micromoth diversity of the arid environments of the central Andes. As this study was restricted to the northern of the two disjunct areas inhabited by *S.philippiana*, further surveys in the southern part of its range would be helpful to assess if the two species collected in the highlands of the Andes are also found in the lowlands of the Atacama Desert.

Generic assignment for a given plume moth species can be a difficult task when it involves some morphologically similar genera of Oidaematophorini, as shown by several species that have moved amongst *Adaina*, *Hellinsia* and *Oidaematophorus* (Gielis 1991, Gielis 2003, Gielis 2011). Phylogenetic analysis of mitochondrial DNA sequences provides a valuable tool in cases like these, as shown in several families of Lepidoptera (e.g. Moreira et al. 2012, Corley et al. 2020, San Blas et al. 2021). In the present study, in agreement with morphology, the result of the phylogenetic analysis provides support for the generic assignment of the two species of Oidaematophorini reared from *S.philippiana*, because each grouped with the type species of its respective genus. However, a clade must have at least 80% SH-aLRT and 95% UFBoot support to be reliable (Minh et al. 2022). Although the SH-aLRT support values for *Adaina* and *Oidaematophorus* are higher than 80%, those of UFBoot are lower than 95%. Accordingly, further phylogenetic analysis, based on wider taxon sampling and additional molecular markers, would be useful to understand better the evolutionary relationships of Neotropical Oidaematophorini and to provide support for delimitation of its genera.

The knowledge of the Neotropical fauna of plume moths has significantly improved in the last thirty years (Gielis 1991, Gielis 2006, Gielis 2011) and recent contributions suggest that many environments of this region harbour additional undiscovered species (Ustjuzhanin et al. 2021a, Ustjuzhanin et al. 2021b, Ustjuzhanin et al. 2021c). As shown in several studies, surveys for adults and immature stages are fundamental to continue the improvement of the understanding of systematics, geographic ranges and host plant use of the plume moths of a given geographic area (Landry and Gielis 1992, Landry 1993,Landry et al. 2004, Matthews et al. 2012, Matthews et al. 2019). Accordingly, field work in under-explored environments should be encouraged to understand better the highly diverse Neotropical fauna of plume moths.

## Supplementary Material

XML Treatment for Oidaematophorus andresi

XML Treatment for Adaina jobimi

## Figures and Tables

**Table 1.** DNA barcode sequences used in the molecular analysis. The asterisk indicates the sequences generated in this study.

**Species**	**BOLD accession**	**GenBank accession**
Oidaematophorini		
*Adainajobimi* Vargas, 2020		OP281685*
*Adainamicrodactyla* (Hübner, [1813])	ABOLA573-14	
*Emmelinamonodactyla* (Linnaeus, 1758)	FBLMT634-09	GU706791
*Hellinsiaosteodactylus* (Zeller, 1841)	ABOLA920-15	
*Oidaematophorusandresi* sp. n.		OP281687*
*Oidaematophorusandresi* sp. n.		OP281688*
*Oidaematophoruslithodactyla* (Treitschke, 1833)	LEATE533-13	
Outgroups		
*Lioptilodesalbistriolatus* (Zeller, 1871)	FBLMS218-09	HM901993
*Platyptiliagonodactyla* (Denis & Schiffermüller, 1775)	FBLMT643-09	GU706667
*Stenoptiliapterodactyla* (Linnaeus, 1761)	FBLMS218-09	HM901993
